# Spontaneous Rupture of an Ovarian Artery Aneurysm: A Rare Postpartum Complication

**DOI:** 10.1155/2016/1029561

**Published:** 2016-02-29

**Authors:** Christopher A. Enakpene, Toni Stern, Marco J. Barzallo Salazar, Pradip Mukherjee

**Affiliations:** ^1^Maternal Fetal Medicine Division, Department of Obstetrics and Gynecology, University of Illinois at Chicago, 820 S. Wood Street (MC 808), Chicago, IL 60612, USA; ^2^Department of Obstetrics and Gynecology, Coney Island Hospital, 2601 Ocean Parkway, Brooklyn, NY 11235, USA

## Abstract

*Background*. Spontaneous rupture of an ovarian artery aneurysm is a rare but usually life-threatening event. It is most often associated with pregnancy or fibroids. Our case followed a normal vaginal delivery and then a delayed presentation with features similar to other less life-threatening postpartum conditions. The diagnosis could have been missed but for the meticulous and timely interventions which avoided catastrophic outcome.* Case*. This is a case of a multiparous woman with rupture of a left ovarian artery aneurysm, causing massive retroperitoneal hemorrhage and hematoma that required a combination of arterial embolization, percutaneous CT scan guided drainage, and surgical evacuation of the hematoma.* Conclusion*. Spontaneous rupture of ovarian artery should be considered as one of the differential diagnoses in the immediate postpartum period especially when the clinical symptoms do not correlate with the amount of blood loss. A high index of suspicion, prompt diagnosis, intervention, and a multidisciplinary approach in the management were the elements of a successful outcome in this case.

## 1. Introduction

Spontaneous rupture of an ovarian artery aneurysm is a rare and usually life-threatening event, most often associated with pregnancy or fibroids [1, 2]. Pregnancy related conditions account for over 50% of ruptured arterial aneurysms in women under the age of 40 years [3, 4]. The hemodynamic and endocrine changes during pregnancy most probably result in arterial alterations, which may lead to new aneurysm formation and/or weakening of preexisting aneurysms. The arteries most commonly affected with aneurysm and rupture during pregnancy are the aorta, cerebral arteries, splenic artery, renal artery, coronary artery, and ovarian artery [3]. Majority of rupture of artery aneurysm usually mimics other less life-threatening disease conditions in its clinic presentation. This can result in delay diagnosis and treatment that may have catastrophic outcomes. Hence, early diagnosis and prompt treatment of a rupture arterial aneurysm are panacea for maximum chances of survival for the mother and baby [3]. The common presentation of spontaneous rupture of an ovarian artery aneurysm is abdominal pain in a multiparous woman during the early postpartum period and the probable time of rupture can be traced to the second stage of labor due to intense pushing [5, 6]. Aneurysms of the ovarian artery have been reported in the literature infrequently [5].

Here we report a postpartum patient with prompt diagnosis of rupture of an ovarian artery aneurysm that was complicated with retroperitoneal hemorrhage and hematoma. Ovarian and uterine artery embolization, minimally invasive drainage, and laparotomy and evacuation of retroperitoneal blood clots are shown to be effective in the management of this case.

## 2. Case

A 38-year-old P4145 with preterm delivery at 34 weeks for her fifth baby was readmitted four days postpartum with complaints of weakness, dizziness, and abdominal pain. On admission, she was anemic, hypotensive, and tachycardic. She also had a palpable abdominal mass in the left flank, which was expanding. The index pregnancy was complicated by hyperthyroidism that was well controlled with Methimazole, chronic left leg varicosity that worsened during pregnancy, multiparity with a cystocele, and depression that was diagnosed during the pregnancy. The patient had an uncomplicated preterm vaginal delivery, a normal immediate postpartum course, and she was discharged home two days postpartum.

**Figure 1:**
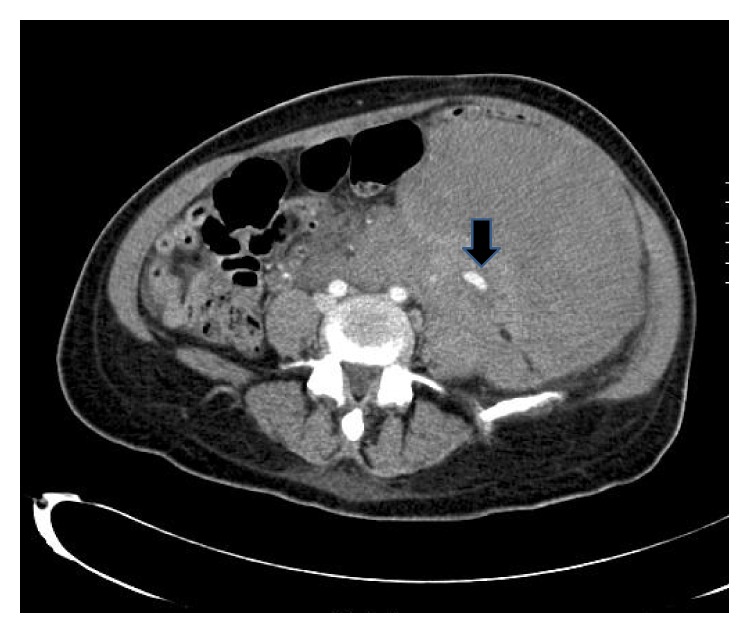
CT axial image with IV contrast: CT axial image of lower abdomen demonstrates 13 mm × 70 mm aneurysm in the left lower quadrant associated with a large hematoma. This corresponds to the aneurysm in the selective left ovarian arteriogram in Figure 3 (arrow).

**Figure 2:**
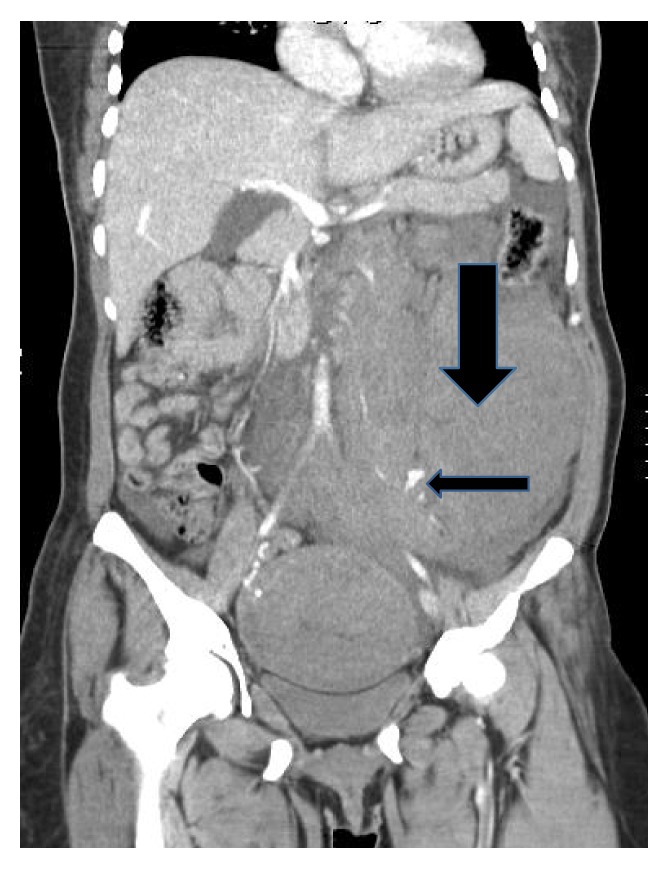
Retroperitoneal hematoma: coronal image of CT of the abdomen shows aneurysm in the left lower quadrant (narrow arrow), associated with a large left lower quadrant hematoma (bold arrow).

**Figure 3:**
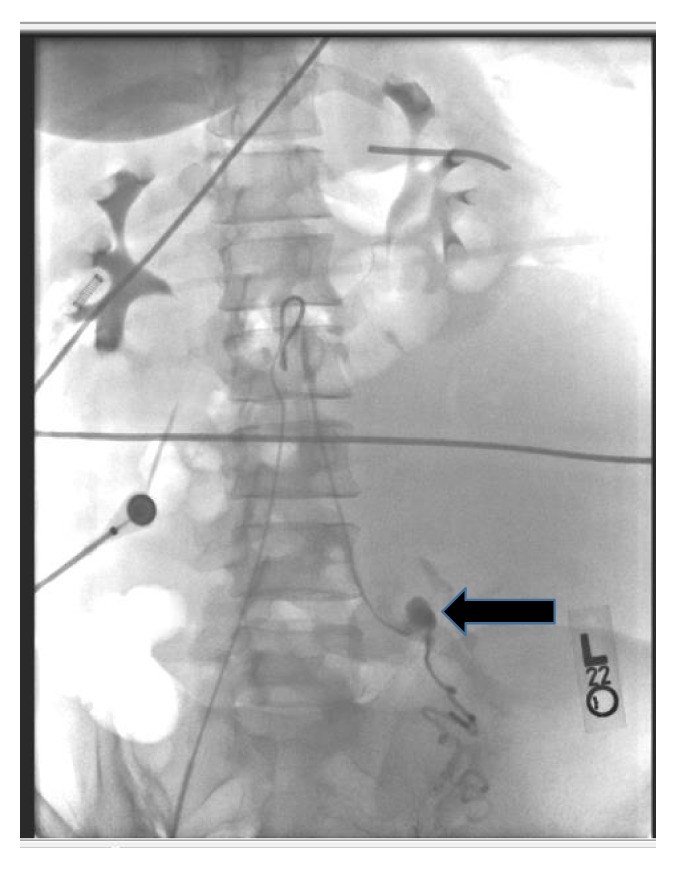
Left ovarian artery aneurysm: selective left ovarian arteriogram demonstrates an ovarian artery aneurysm (arrow).

**Figure 4:**
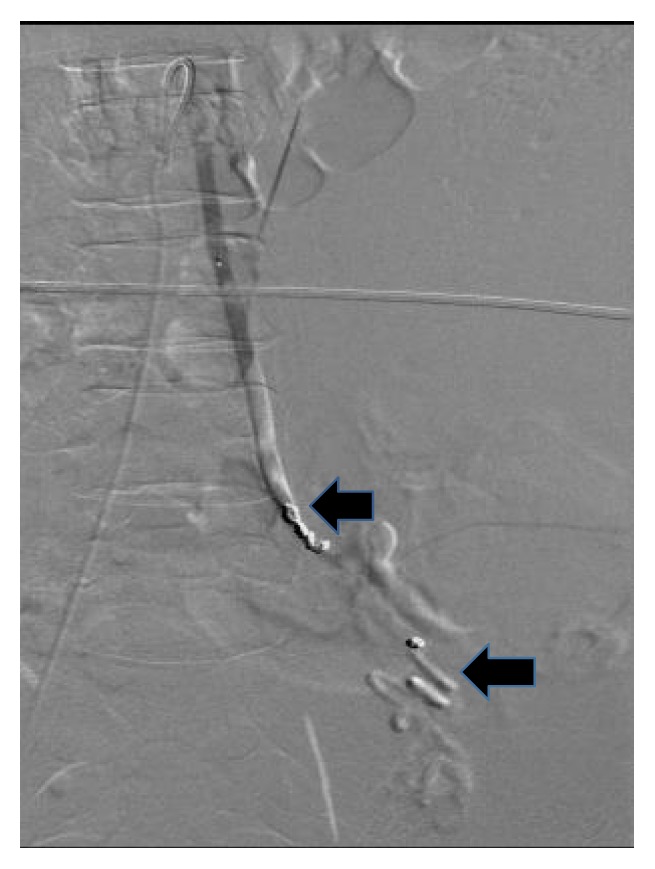
Post-left ovarian artery embolization: this shows microcoils placed proximal and distal to the aneurysm (arrows).

**Figure 5:**
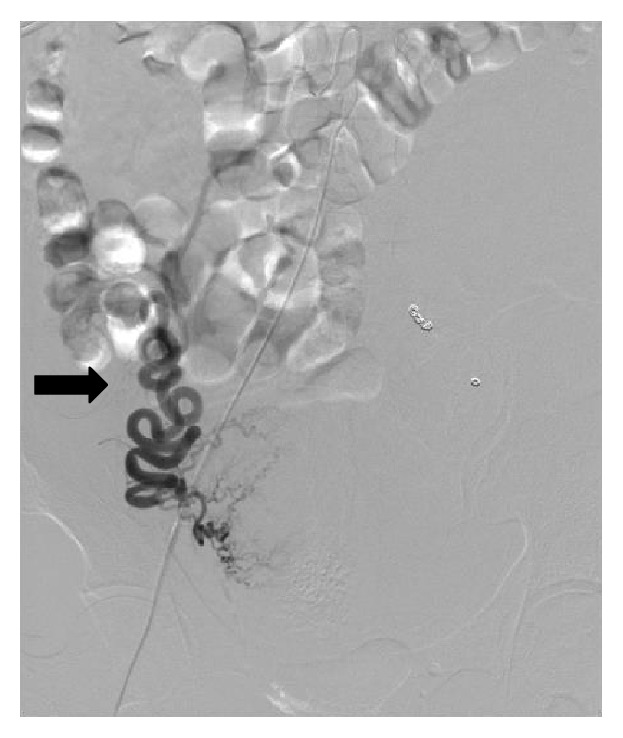
Right ovarian artery angiogram: markedly dilated and tortuous right ovarian artery with distal branches supplying right side of the uterus (arrow).

At readmission, her hemoglobin/hematocrit were 8.1/24.0 as compared to 11.1/32.7 at discharge two days priorly. A CT scan of the abdomen and pelvis with contrast demonstrates a large retroperitoneal hemorrhage due to a ruptured left ovarian artery aneurysm (Figures 1 and 2). She underwent selective left ovarian artery angiogram and embolization using microcoils and Gelfoam pledgets (Figures 3 and 4) drainage of retroperitoneal hemorrhage. However, her hemoglobin levels did not improve significantly despite aggressive resuscitation with multiple transfusions of blood products. On the 2nd day, she had a repeat CT scan of the abdomen and pelvis with contrast, which showed increase in the size of the hemorrhage and hematoma on the left side and a tortuous and dilated right ovarian artery (Figure 5). She subsequently had prophylactic embolization of the right ovarian artery and the uterine arteries bilaterally. The patient received a multidisciplinary care approach in the Surgical Intensive Care Unit involving teams from interventional radiology, surgery, medicine, endocrinology, OB/GYN, and hematology. She received a total of 8 units of packed red blood cells (PRBC), 3 units of fresh frozen plasma (FFP), and 1 unit of platelets throughout the course of treatment. The hyperthyroidism was managed with Methimazole, potassium iodide, and propranolol. By the 4th day on admission, a total of 1500 mL of retroperitoneal hematoma have been drained through the drain placed by the interventional radiology. However, due to persistent abdominal pain and development of fever, which was not responding to high potency antibiotics, she had exploratory laparotomy and surgical evacuation of the remnant hematoma. The patient improved remarkably after the laparotomy and she was discharged home four days post-op in a stable condition.

The patient has resumed normal activities and she has remained asymptomatic since discharge.

## 3. Comment

Ovarian aneurysmal rupture causing severe abdominal pain is a rare event with only a few documented cases in literature. When evaluating a patient with abdominal pain, it should be considered in the differential diagnosis. Previous cases of ovarian aneurysm have mostly been treated with embolization or surgery [7, 8]. However, there is currently not enough data to determine the ideal approach and effective treatment to ovarian artery aneurysmal ruptures. This rare condition can result in catastrophic hemorrhage, serious morbidity, and even death without prompt diagnosis and immediate intervention. There is a high index of suspicion in women with risk factors whose blood loss is not commensurate with their clinical scenarios and this should prompt early imaging studies for diagnosis and urgent treatment. Majority of previously reported ovarian artery aneurysms usually present with spontaneous rupture in the puerperium [9–11], especially in the immediate postpartum periods [12]. It has also been reported in nonpregnant patients [1] and sometimes during the antepartum period [13]. Very few asymptomatic cases are incidentally identified during evaluation for other reasons [14]. Unlike other previously reported cases of ovarian artery aneurysm, this is the first report of bilateral ovarian artery aneurysm with rupture of one of the aneurysms that presented a few days after delivery. This is also the only reported case that required a combination of arterial embolization, interventional radiology drainage, and subsequent surgical evacuation of the hematoma. The use of interventional radiology drainage as a first line of management was due to the fact that the ruptured ovarian artery aneurysm resulted in a large retroperitoneal hemorrhage, which could be drained using minimally invasive approach at the same time of arterial embolization. The drainage was very effective initially and drained 1500 mL within 48 hours of the CT guided placement of drainage tube. The clots became organized requiring surgical evacuation later in the course of her management.

## 4. Conclusion

Prompt diagnosis and effective supportive therapy are required for the management of rupture ovarian artery aneurysm. As in previous cases, adequate embolization in addition to percutaneous drainage and surgical evacuation played important role in the treatment for this patient.
